# A Review of Murine Cytomegalovirus as a Model for Human Cytomegalovirus Disease—Do Mice Lie?

**DOI:** 10.3390/ijms22010214

**Published:** 2020-12-28

**Authors:** Michelle A. Fisher, Megan L. Lloyd

**Affiliations:** 1 Division of Infection and Immunity, School of Biomedical Sciences, The University of Western Australia, Nedlands 6009, Australia; michelle.fisher@uwa.edu.au; 2 Marshall Centre for Infectious Diseases Research and Training, Division of Infection and Immunity, School of Biomedical Sciences, The University of Western Australia, Nedlands 6009, Australia

**Keywords:** congenital disease, placenta, salivary gland

## Abstract

Since murine cytomegalovirus (MCMV) was first described in 1954, it has been used to model human cytomegalovirus (HCMV) diseases. MCMV is a natural pathogen of mice that is present in wild mice populations and has been associated with diseases such as myocarditis. The species-specific nature of HCMV restricts most research to cell culture-based studies or to the investigation of non-invasive clinical samples, which may not be ideal for the study of disseminated disease. Initial MCMV research used a salivary gland-propagated virus administered via different routes of inoculation into a variety of mouse strains. This revealed that the genetic background of the laboratory mice affected the severity of disease and altered the extent of subsequent pathology. The advent of genetically modified mice and viruses has allowed new aspects of disease to be modeled and the opportunistic nature of HCMV infection to be confirmed. This review describes the different ways that MCMV has been used to model HCMV diseases and explores the continuing difficulty faced by researchers attempting to model HCMV congenital cytomegalovirus disease using the mouse model.

## 1. Introduction

It has famously been stated that “mice lie and monkeys exaggerate” [1], but the use of animal models in the study of infectious disease provides strong evidence for the mechanisms underlying the pathogenesis of infections in humans and provides opportunities to study interactions between hosts and pathogens that are not provided by collecting minimally invasive human samples such as blood, feces, saliva and urine. When investigating infectious diseases, the ability to correlate tissue histopathology with pathogen replication rates over time or evaluate cell signaling and related immune modulation in the context of a complete immune system is of inestimable value and provides mechanistic information, as well as pre-clinical opportunities to test treatment efficacy and toxicity. The ability of a pathogen to infect both mice and humans allowed the development of Koch’s postulates [2], an evidence framework still used today to prove the association of newly described pathogens with infection and subsequent pathological sequelae, albeit with modifications to account for the presence of pathogens in commensal flora [3] and viruses that are attenuated in a culture [4]. An example of this modern reasoning has been applied for SARS-CoV-1 [5].

There are many animal models where laboratory-inoculated animals exhibit symptoms or pathogen replication that mimics human infection, even though infection may not occur in the natural environment. These models are useful in carefully evaluated (and disclosed) circumstances (discussed in [6] for SARS-CoV-2) and have provided useful information on infection pathways and the effects of opportunistic infections and potential drug targets [7]. Examples of animal models in non-target species are those developed for influenza and respiratory syncytial virus in ferrets [8] and Ebola virus infection, which have been modeled in suckling (but not adult) immunocompetent mice as well as Syrian golden hamsters [9]. These models and others like them have illuminated pathways used by pathogens to travel from the site of infection to organs or other sites of tropism, as well as immune responses, symptom and infection profiles and treatment options.

In animal models for viral disease, the presence of the correct receptor used by the virus to enter a cell is critical and needs to be largely conserved between species for cross-species infection to occur [6]. For infections where viruses do not naturally infect another species due to absence of the correct receptor, transgenic mice have been developed that allow an animal model to be developed (e.g., for poliovirus) [10]. Some viruses, such as influenza, naturally infect multiple species (avian species, pigs and humans [11]) while others, such as cytomegalovirus, are species-specific. Animal models where the pathogen naturally infects the animal host and the disease is naturally present in wild populations can provide insights into human disease by illuminating complex host-pathogen interactions and provide clinically relevant data.

Cytomegaloviruses (CMVs) from the family Herpesviridae and the subfamily Betaherpesvirinae are species-specific viruses and have been isolated from many mammalian species (e.g., human, mouse, rat, guinea pig and various primates; see [12] for a discussion). CMVs have evolved with their hosts [13] and generally do not replicate completely in vivo in different species after inoculation, even where in vitro growth has been described (e.g., murine cytomegalovirus (MCMV) will grow in vitro in rat cells but will not grow in vivo in rats [14]). However, there have been reports of the replication of disparate CMV in other species in circumstances where xenotransplantation has taken place and the recipient has received immunosuppressive chemotherapy [15,16]. Juvenile rats have also been reported to support the growth of MCMV, although older rats (>6 weeks old) were not susceptible to infection [17]. This review will focus on the use of MCMV to model human cytomegalovirus (HCMV) disease.

## 2. Murine Cytomegalovirus Infection as a Model for Human Cytomegalovirus Disease

MCMV infecting its murine host has been used extensively to model human disease with HCMV [18]. MCMV is a natural pathogen of mice, being found in every wild *Mus musculus* population that has been investigated in Australia (for example, those found in [19,20]). There are many genetic similarities between HCMV and MCMV with multiple gene homologs, including structural and immune-evasion genes [21]. However, there are important differences in the organization of the genetic information. For example, HCMV has unique long and unique short regions with terminal and internal repeat sequences [22] compared with MCMV, which has a single unique sequence with short terminal direct repeats and several short internal repeats [21]. Additionally, the adaptation of these viruses to cell culture is different, as the in vitro culture of clinical isolates of HCMV invariably leads to the predictable loss of genetic regions (discussed in [23]) that does not occur to the same extent with MCMV [24], although a recent report has provided evidence that deletions occur as MCMV is propagated from salivary glands in culture [25].

### 2.1. Properties of Salivary Gland-Derived and Tissue Culture-Passaged MCMV

Modeling HCMV infection with MCMV with the use of virus stocks prepared from different sources is complicated. MCMV is generally maintained either as salivary gland stock (SGV) or as tissue culture-derived stock (TCV), usually prepared from the infection of embryonic mouse fibroblasts [26]. This influences the nature of the infection that occurs in vivo [27]. SGV is generally composed of single capsid virions, derived from the cytoplasmic vacuoles within the serous acinar sinus cells of the salivary gland [28]. It produces acute infection in mice, is lethal when inoculated at a relatively low titer in juvenile (3-week-old) mice and has been used in Lethal Dose_50_ (LD_50_) studies to demonstrate innate major histocompatibility (MCH) associated resistance to MCMV in various inbred mouse strains [29,30]. In contrast, virus stocks made from other organs (e.g., the liver and spleen) and also TCV passaged in embryonic mouse fibroblasts are comprised of both single and multicapsid virions [31]. TCV is generally not lethal, unless the mouse strain has a substantial immune deficiency (e.g., severe combined immuno-deficient mice that do not have B or T cells can be lethally infected with 1 plaque-forming unit (PFU) of SGV [32]).

### 2.2. MCMV as a Model for HCMV Infection

The recorded movement of MCMV between organs differs, depending on the route of infection (described in [33] and [12]). There is a strong tropism for the salivary gland, and active viral replication persists in salivary glands for longer than other organs, regardless of the route of infection used. As with all herpesviruses, MCMV infection has a latent phase [32]. MCMV infection without significant manipulation of the host has successfully been used to model various aspects of human infection (Table 1). Information from this natural animal model has greatly improved the understanding of the pathogenesis of HCMV infection in humans.

### 2.3. Mouse Strain Selection Affects the Severity of MCMV Pathogenesis

Investigations of the effect of MCMV in different mouse strains have been pivotal in the modeling of different disease states. Initial work focused on LD_50_ calculations (e.g., [46]), and it was found that the H-2 alleles of different mouse strains determined their response to infection, including the production of autoantibodies [47] and the induction of myocarditis [48]. Other mechanisms for resistance to MCMV, such as that demonstrated in C57BL/6 mice compared with BALB/c mice or the differing resistance of New Zealand Black and White mice to MCMV infection, were associated with differences in innate natural killer (NK) cell activation and were strongly associated with particular strains of viruses [49,50]. Where MCMV was used as a vaccine vector expressing the mouse ovarian glycoprotein zona pellucida 3 in studies investigating immune-mediated contraception, the specific m157 (viral ligand) to Ly49H (NK cell activation receptor) rapid response to infection was broadly associated with vaccine success [51]. This effect was abrogated through the use of a different virus strain, G4 (isolated from the salivary glands of a mouse from Geraldton, WA [52]), as the vaccine vector. G4 does not have the same interaction with NK cell activation receptors, and this demonstrates the importance of vector strain selection in the development of recombinant CMV-based vaccines [53].

### 2.4. MCMV-Based Models for Human Disease Requiring Chemical, Genetic or Physical Manipulation

A recent systematic review and metanalysis calculated that the worldwide seroprevalence of HCMV is 83% [54]. Increasing CMV disease is broadly associated with improvements in medicine because it is often associated with acute immunosuppression, allowing the reactivation of a latent infection. One of the more serious sequelae of CMV infection is found in solid organ transplant recipients, who often experience reactivation from latency and associated pneumonitis, hepatitis and potential organ rejection (discussed in [55]). The likelihood of severe CMV disease increases when the transplanted organ is from a seropositive donor (previously infected with CMV, with no active viral replication but a strong CMV-specific humoral response) being transplanted into a seronegative recipient [56]. In general, outcomes can be improved with the use of antiviral therapy, with a recent metanalysis suggesting that prophylactic treatment using low doses of valganciclovir provides improved outcomes in kidney transplant recipients [57]. CMV can also cause post-transplantation disease in recipients of allogeneic hematopoietic stem cell transplants, and pre-emptive therapy is often initiated after clinical evidence of CMV reactivation (prior to fulminant disease). The economic burden of this therapy is marked [58]. The mouse model of MCMV has been integral in the prediction of useful therapeutics for these clinical circumstances (reviewed in [59], with a discussion of the appropriate use for this model in reliably predicting human outcomes).

In order to model CMV-associated diseases occurring due to immunosuppression, such as retinitis or post-transplantation reactivation, the animal model needs to be manipulated to ensure similarity to human infection. The modification can be due to chemical administration (e.g., corticosteroid use), genetic modification of either the virus or the mouse strain used (e.g., MCMV-deleted m157 virus (Δ157) allows C57BL/6 mice to be used without NK cell activation by the virus) or physiological treatment such as surgery. These modified models are listed in Table 2.

### 2.5. MCMV Exacerbates the Effects of Other Clinical Diseases

For some models, the addition of MCMV can exacerbate disease, reflecting human disease particularly in the intensive care unit setting. These are generally diseases with an immune modulation component. These models are listed in Table 3.

### 2.6. Routes of Infection

Many early CMV diseases were modeled using the intraperitoneal route of infection. The movement of a virus from the site of vaccination and the dissemination of a virus via peritoneal macrophages to visceral organs and, finally, to the salivary gland has been well described [60]. This route of inoculation does not correlate well with natural routes of infection, and other inoculation sites, such as the footpad, have also been used in some iterations of the MCMV disease model. Inoculation of a virus via the footpad (also referred to as intrafoot) is thought to model transmission by biting, and the infectious virus moves to the popliteal lymph node, infecting resident subcapsular sinus macrophages, although these cells do not allow lytic replication. Viremia and further organ infection may be due to a cell-free virus [61]. Recently, the intranasal route of inoculation (simulating transmission from mother to offspring via grooming) has been investigated more rigorously, and this has demonstrated that the visceral organs do not show the same level of viral replication as is seen with other routes of administration [62]. When investigated using a fluorophore-labeled virus, it was demonstrated that infection was via the olfactory neurons and disseminated by day 16 after inoculation to the salivary gland [63]. The MCMV/mouse model has recently been used to show temporal differences in virus dynamics and that there is interplay between different viral strains. Importantly, this work suggests that an increased number of virus strains present in one mouse is not indicative of more substantial pathological changes [64].

### 2.7. Humanized Mouse Models

The manipulation of mice to allow humanized features allows infection with HCMV [98] and increases the repertoire of testing available for HCMV preventative strategies, as well as pre-clinical testing of treatments such as chemotherapeutic interventions. For example, humanized immune system mice have been investigated for future use in modeling vaccine efficacy [99]. Although this strategy provides additional information, the validity of the results needs to be carefully evaluated in every model, particularly with respect to the immune responses generated in the mouse.

### 2.8. Immunology and MCMV Studies

This is a large topic and will not be reviewed extensively here, although many fundamental discoveries have been possible due to the robust nature of this murine model. Research using MCMV has allowed specific viral immune defense processes to be elucidated and for basic research investigating the activity and effector functions of immune cells to be undertaken. These studies have provided important insights into defense from lifelong HCMV infection, including the interesting phenomenon of memory inflation, where a disproportionate percentage of the CD8+ T cell response is devoted toward HCMV immunity [100]. Immune responses to MCMV have been recently reviewed [101].

#### 2.8.1. Innate Immune Responses

Innate immune responses determine the severity of infection, and NK cells play an important early role in defense from MCMV infection. Research in this area (originally undertaken to describe the difference in LD_50_ between different mouse strains [102]) has demonstrated the presence of NK cell activating receptors and the antigen-specific inflation of NK cell populations [103], responsible for the increased resistance of C57BL/6 mice to MCMV infection compared with BALB/c mice [104]. Altered NK cell development and the development of long-lasting NK cell memory has been demonstrated using the mouse model (reviewed in [105]), altering the perception of NK cells as only contributing to innate immune processes. Recent evidence that infection via the intranasal route infects restricted cell types suggests that the route of infection needs to be carefully assessed in these models [106]. Cytokines produced as a result of MCMV infection are responsible for tissue damage, as well as suppression of pathology, and were discussed in detail in a recent review [107].

#### 2.8.2. Adaptive Immune Responses

Strong humoral and cell-mediated immunity is induced after primary infection with MCMV, with CD8+ T cells being important for the control of viral clearance from many organs [108] and having recently been confirmed to inhibit MCMV-associated effects on hematopoiesis in bone marrow graft rejection [109]. The importance of CD4+ T cells in viral clearance is well established [108], and more recent reports have shown that CD4+ T cells produce granzyme B and may directly kill infected cells in vivo [110]. Antibody responses to viral infection were characterized in different mouse strains in early research, which showed that autoantibodies were produced as part of the response to primary infection [47] and were due to polyclonal B cell responses [111]. Administration of a passive antibody reduces MCMV organ titers but does not prevent infection [112]. However, recent data suggests that the administration of an antibody toward glycoprotein B, even where the antibody is unable to neutralize the virus, may provide protection from disease [113].

### 2.9. Congenital Infection with Cytomegalovirus

One of the aspects of HCMV infection that has repeatedly been attempted using various manipulations of the basic MCMV/mouse model is congenital infection. In humans, symptomatic CMV infection is present in 0.07% of births [114]. The most serious sequelae are associated with primary maternal infection during the first trimester of pregnancy, including still births [115] as well as disseminated CMV inclusion disease [116]. If the transmission occurs later in pregnancy, the risk of transmission is greater (around 40% and 70%, respectively), but the risk of the most serious sequelae is diminished [117]. Infection outcomes have a spectrum of symptoms, ranging from pneumonitis, hepatitis and growth restriction to mental retardation, impairment in motor function and escalating sensorineural hearing loss (SNHL) or, alternatively, children can remain clinically normal [118]. Congenital CMV (cCMV) infection is diagnosed using a combination of maternal serology, ultrasonography and amniocentesis, and the severity of disease cannot always be predicted with accuracy [119]. Virtually as soon as MCMV was isolated, attempts were made to model congenital disease, mostly with frustrating outcomes [120]. Experimental manipulation of the mouse/MCMV model to simulate human cCMV disease is shown in Table 4. To date, the most successful model of congenital disease is the guinea pig model (discussed in [121]). However, the disease is generally acute, and there are limitations with reagent availability and the lack of inbred lines for experimentation. Guinea pigs also have a much longer gestation period (around 65 days, compared with 21 days for mice) and often have three or fewer pups.

#### 2.9.1. Mouse Models of Congenital Infection

Early studies of cCMV used relatively high titers of SGV and generally resulted in fetal loss (resorption) and growth restriction that was attributed to maternal illness or placental insufficiency [122,125]. It is important to note that the mice in these early studies were not likely to be specific pathogen free and undetected co-infections may have affected these data. Experimental maternal infection with MCMV was often associated with smaller placentae, and although placental infection was reported by some researchers, transmission to the fetus was rarely detected, and resorption of fetuses was common. This suggested that fundamental differences between the mouse and human placentae may be responsible.

#### 2.9.2. Comparative Anatomy of Mouse and Human Placentae

**Figure 1:**
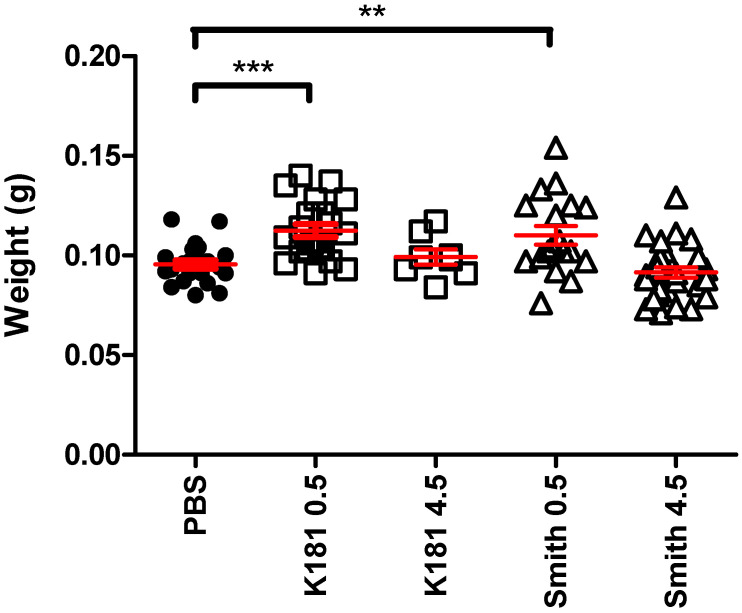
Effect of MCMV infection during pregnancy on placental weight (placental weights of E18.5 fetuses inoculated on day 0.5 or day 4.5 of pregnancy). Female BALB/c mice (*n* = 5) were infected with 2 × 10^4^ PFU MCMV (K181_Perth_ or Smith strain TCV) or the same volume of phosphate buffered saline (PBS) on the day of fertilization (E0.5) or on the day of blastocyst implantation (E4.5, viruses only) via the intraperitoneal route. Placentae were collected on E18.5 and weights were recorded. Mean ± SD are shown in red. ** *p* < 0.01. *** *p* < 0.001, ANOVA, Tukey’s multiple comparison test.

There are many similarities between the placentae of humans and mice, with the most notable being that both are hemochorial (i.e., having a trophoblast surface that is in direct contact with maternal blood). The most obvious difference, and one that probably results in reduced fetal infection, is that mice have three trophoblast layers—one is mononuclear and two are syncytial—separating the maternal and fetal blood, compared with the single syncytiotrophoblast layer found in human placentae [137,138]. The three-cell layer has previously been theorized to act as a physical barrier to the transmission of MCMV [122]. However, the reported congenital infection of severe combined immunodeficient (SCID) mouse pups leading to intrauterine growth restriction and microcephaly suggested that this physical barrier may not be responsible for differences between mice and humans [129]. The use of TCV rather than SGV has allowed some more subtle infections to be modeled, particularly involving infection of the placenta without significant resorption of fetuses (Figure 1). Female mice were mated and inoculated i.p. with MCMV TCV stock, either K181_Perth_, the origins of which have been recorded previously [52], or the Smith strain [139] on either E_0.5_ (the day of fertilization) or E_4.5_ (the day of implantation), and placentae were collected on E_18.5_. The placentae were weighed (UWA Animal Experimentation Ethics Committee approval 100/788). Figure 1 demonstrates that the placental size was significantly increased in mice infected on the day of fertilization compared with those infected on the day of implantation. Increased placental thickness is a hallmark of cCMV disease in humans [140].

#### 2.9.3. Mouse Brains Are Refractory to MCMV Infection

Mouse embryonic stem cells have been reported to be refractory to MCMV infection, but neuronal stem progenitor cells are susceptible to infection, with brain infection occurring particularly at the cerebral subventricular zone [141]. Experiments using brain slice cultures have shown that the susceptibility of brain cells, particularly neural stem progenitor cells, to CMV infection may be associated with neurogenesis (reviewed in [142]). The susceptibility of newborn pups to brain infection after i.p. inoculation has been described, and the protection afforded by CD4^+^ T cells [143], CD8^+^ T cells [144], and antibodies [130] has been noted. This model has provided important information about sensorineural hearing loss [132]. Latent MCMV in brain tissue has been reactivated in brain slices in a culture from mice infected as newborn pups [145].

## 3. Conclusion—Do Mice Lie?

The MCMV/mouse model is a robust example of an animal model that should be used with a clear understanding of the differences between the human and mouse viruses, their adaptation to cultures and physiological differences between species, including differences in immune responses. MCMV has been very useful in modeling broad aspects of HCMV disease in many different situations, now greatly expanded with the use of recombinant viruses and genetically modified mouse strains. This has resulted in many different clinical conditions being accurately simulated and has allowed the opportunistic nature of HCMV, including its effect on the exacerbation of unrelated diseases, to be demonstrated. The only type of disease that is consistently difficult to replicate is congenital disease, due mostly to fetal resorption associated with acute maternal disease and potentially exacerbated by differences in placental structure and in the receptivity of the brain to MCMV replication. However, the use of TCV rather than SGV and the detection of viruses by means other than cultures, as well as careful evaluation of neonatal mouse inoculation, has provided some exciting alternative models with predictive capacity. Identifying the subtle differences in experimental procedures used by different researchers will improve experimental plans and aid in the appropriate interpretation of results.

## Figures and Tables

**Table 1:** Human disease caused by human cytomegalovirus (HCMV) modeled by murine cytomegalovirus (MCMV) infection in mice.

Human Condition	Lab Conditions	SGV ^1^/TCV ^2^ PFU ^3^	Effect	Reference
Viremia	Intraperitoneal (i.p) inoculation of BALB/cByJ mice.	SGV/TCV not specified 10^6^ PFU	White blood cells have viral DNA but no evidence of *ie1* RNA.	[34]
Viral latency	BALB/c footpad inoculated at 2 weeks of age. Latency present after 3 weeks.	TCV 10^5^ PFU	Whole body irradiation leads to reactivation of infection. Antibody protects from viral dissemination.	[35,36]
Pneumonitis	1. Intranasal inoculation into outbred Swiss mice or intra-tracheal infection of BALB/c mice.	TCV >10^4^ PFU	Severe diffuse interstitial pneumonitis closely resembling that seen in immunocompromised patients and in newborn infants, 20% died.	[37]
	2. Inoculation of newborn BALB/c.	SGV, 6 PFU i.p.	Pneumonitis and myocarditis, 95% lethal.	[38]
Hepatitis	i.p inoculation of BALB/c mice.	SGV, 10^5^ PFU	Hepatitis evident, dose is Lethal Dose_50_.	[26]
Ocular infection (retinitis)	1. Intraocular inoculation (scarified cornea or through corneal limbus) of IRC/Sic mice. 2. Intraperitoneal inoculation of BALB/c mice.	SGV, Tissue Culture Infectious Dose_50_ values given.	Different effects SGV vs. TCV.	[39]
			Inflammatory response in retina (virus not present) and iris (virus present).	[40]
Excretion of CMV into breastmilk	Acute or latently inoculated C57BL/6 mothers (i.p.), leukocytes from BM positive by *ie1* mRNA detection, RTPCR.	SGV 3 × 10^2^–3 × 10^4^ PFU	Evidence of neonatal infection via breast milk. Inoculation of milk into CD-1 1-day old mice results in infection.	[41]
Arterial blood pressure post CMV infection	i.p. inoculation of 2-week-old C57BL/6 mice.	TCV, 3 × 10^5^ PFU/1mL/mouse	MCMV increased blood pressure independent of diet. Increased serum IL-6, TNF-alpha, and MCP-1.	[42]
Viral myocarditis	i.p. inoculation of C57BL/6 and BALB/c mice.	SGV 10^4^ PFU	Inflammatory foci in the heart and infection of cardiac myocytes.	[43]
Infection post bone marrow transplantation	Irradiated BALB/c mice inoculated with virus prior to intravenous (i.v.) purified bone marrow cells.	10^5^ PFU	Failure in haematopoiesis, leading to death.	[44]
Sexual transmission of CMV via semen	Spermatozoa plus Smith MCMV artificially inseminated (compared with sperm alone).	SGV 10^5^ PFU	Embryos collected on E14. One produced cytopathic effect (second passage). No significant difference with numbers or abnormalities.	[45]

^1^ Salivary gland derived virus. ^2^ Tissue culture-derived virus. ^3^ Plaque-forming units.

**Table 2:** Models for CMV disease, requiring significant laboratory manipulation.

Human Condition	Lab Conditions	SGV ^1^/TCV ^2^ PFU ^3^	C ^4^	G ^5^	P ^6^	Effect	Reference
Reactivation after immunosuppression	i.p. inoculation of C3H/St mice.	SGV subcutaneous infection with 10^3^ PFU Latency = 8 months	x			Immunosuppression by rabbit antilymphocyte serum and corticosteroid.	[65]
Pneumonitis	Intranasal MCMV plus cyclophosphamide 24 h after viral inoculation in BALB/c.	SGV, 10^5^ PFU	x			Interstitial pneumonitis.	[66] Reviewed in [67]
Atherogenesis	MCMV inoculated i.p. in APO-E−/− mice (C57BL/6 background).	SGV 10^5^ PFU Or 3 × 10^4^ PFU TCV		x		Larger atherosclerotic lesions in infected mice, potentially caused by upregulation of p38.	[68,69]
Transplant-arteriosclerosis	Human peripheral blood leukocyte/Rag-2−/−γc−/− mouse-xenograft-model inoculated with HCMV.	Segments of mammary artery incubated in vivo with 10^5^ PFU HCMV before implantation		x	x	Transplant arteriosclerosis was significantly elevated and increased ICAM-1, PDGF-R-b and macrophages.	[70]
Reactivation of CMV infection post organ transplantation	Immunosuppression of BALB/c mice receiving donor kidney (C57BL/6 allograft) leads to dissemination of reactivated MCMV.	SGV 10^7^ PFU MCMV-Δm157. Mice used as donors 4–8 months post i.p. inoculation (latency)	x		x	Two-step process: allograft ischemia and reperfusion injury (step 1). Immunosuppression mediated viral dissemination (step 2).	[71]
Sjogren’s syndrome	1. MCMV inoculated i.p. in *Fas*-deficient C57Bl/6-lpr/lpr mice. 2. MCMV inoculated i.p. in tumor necrosis factor-related apoptosis-inducing ligand deficient BALB/c mice.	SGV, 10^5^ PFU SGV 10^4^ PFU		x x		Salivary gland inflammation and autoantibody production. Autoantibody production and lymphocytic aggregates.	[72]
Reactivation of CMV infection post haemopoietic cell transplantation	1. BALB/c mice undergo sublethal irradiation (6 Gy), undergo syngeneic haematopoietic stem cell transplant 6 h later, 2 h later inoculated with CMV via foot pad.	TCV 10^5^ PFU			x	Pulmonary infection control depends on CD8 T cell reconstitution.	[73]
	2. CD8 T cell immunotherapy.					Controls CMV associated immunopathology.	[74] Reviewed in [59]
Graft versus host disease	1. Inoculated with MCMV 3 days prior to transplant. A variety of H-2 defined mice inoculated i.v. with spleen cells (either MHC1 or MHCII disparity). 2. Strain specific antibody therapy. Latently inoculated mice (i.p).	1 × LD_50_ SGV		x		Reduction of CTL and immunodeficiency induced, 10 × less donor cells required.	[75]
		10^4^ (B6 or B6D2F1) or 5 × 10^3^ PFU (BALB/c) SGV		x		Strain-specific antibody therapy protects from MCMV reactivation.	[76]
CMV retinitis	1. Inoculation into supraciliary space (described in [77]) + immunosuppression of BALB/c mice via methylprednisolone every 3 days starting 2 days prior to inoculation.	SGV 5 × 10^3^ PFU	x			Retinitis abrogated using i.v. siRNAs directed against MCMV immediate early protein-3 (IE-3).	[78,79]
	2. Immunosuppression using C57Bl/6J intraretinal inoculation PLUS MAIDs.	SGV 10^4^ PFU	x			(MAIDS—retrovirus mixture defined in [80]) Severity of effect may be due to suppressor of cytokine signaling (SOCS) 1 and 2.	[81]
Renal allograft loss due to MCMV reactivation (donor positive)	Donor BALB/c recipient C57Bl/6 after renal transplantation.	TCV MCMV or Δm157, Infection with 10^4^ PFU (Δm157) all donors. Recipient either 10^4^ PFU Δm157/WT or 10^2^ Δm157.		x	x	Th17 inhibition reduced injury to graft.	[82]
Brain infection in immunosuppressed patients	Severe combined immunodeficient (SCID) mice (BALB/c background), intracranial infection. Virus expressing green fluorescent protein (GFP).	TCV, 10^6^ PFU (GFP), 4.83 × 10^5^ PFU wildtype		x	x	Adoptive transfer of MCMV-specific CD4 T cells clears CMV from the brain. Treatment prior to infection prevents MCMV replication.	[83]
Hemophagocytic lymphohistiocytosis	IFN-γ-knockout (KO) mice on BALB/c background or BALB/c	SGV, 5 × 10^3^ PFU		x		Severity not associated with titer, associated with inflammation.	[84]
Laboratory diagnosis of hepatitis	i.v. inoculation of C57BL/6 mice with marker virus (luciferase, mCherry, SINFEKL)	TCV, 10^6^ PFU		x		Blood biochemistry levels given (allowing diagnosis of hepatitis).	[85]

^1^ Salivary gland-derived virus. ^2^ Tissue culture-derived virus. ^3^ Plaque-forming units. ^4^ Chemical modification e.g., chemotherapeutic. ^5^ Genetic modification of mouse or virus. ^6^ Physiological treatment (e.g., surgery).

**Table 3:** Models demonstrating MCMV-associated effects on other diseases.

Medical Condition	Lab Conditions	SGV ^1^/TCV ^2^ PFU ^3^	Effect	Reference
Ulcerative colitis in CMV-inoculated individuals	T cell receptor alpha−/− mice (C57BL/6 background) inoculated i.p. at 7 days of age with MCMV or MCMV-enhanced GFP	TCV, 5 × 10^5^ PFU of MCMV for C57BL/6 mice and 2 × 10^4^ PFU of MCMV-EGFP for TCR-alpha−/−	Ulcerative colitis is exacerbated in latently infected mice	[86]
Growth of glioblastoma	Mice inoculated i.p. day 2 of life (P2), tumor injection week 15	TCV 10^3^ PFU MCMV-Δm157	Tumor growth and reduction in survival	[87]
CMV reactivation after physical damage	Caecal ligation and puncture in latently i.p. inoculated BALB/c mice	TCV, 2 × 10^4^ cgrmPFU	Plus *S. aureus* to induce bacterial pneumonia	[88,89]
MCMV infection after cholestasis	C57BL/8 mice bile duct ligated, inoculated i.p. Δm157-MCMV-luciferase	SGV/TCV not specified. 2 × 10^5^ PFU	Impaired inflammatory response, but no increase in liver pathology	[90]
Melanomas growth, repeated injection recapitulates transient response	Intra-tumoral inoculation of MCMV or ΔgL MCMV (spread deficient) impairs melanoma growth in BALB/c mice	TCV (described in [91]), 5 × 10^5^ PFU	Infection of macrophages leads to proinflammatory M1 state	[92]
Idiopathic pulmonary fibrosis	Latent MCMV infection in BALB/c mice (i.p.—4 weeks prior), intratracheal bleomycin	SGV, 10^5^ PFU	MCMV-exacerbated fibrosis, activation of TGF-β1	[93]
Experimental autoimmune encephalomyelitis (EAE)	C57BL/6J and CD80/86−/− mice inoculated with MCMV i.p. and 8 days later, EAE induced	SGV, 5 × 10^4^ PFU	More severe disease (e.g., enhanced demyelination), severity associated with number of splenic CD4^+^CD28^null^ T cells	[94]
Use of bronchiolar lavage (BAL) to detect reactivation of CMV in sepsis	BALB/c mice inoculated with MCMV, sepsis by caecal ligation and puncture	SGV/TCV not specified; 10^2^, 10^6^	qPCR of BAL cell pellets similar to that of lung tissue	[95]
Acute colitis	C57BL/6 mice	TCV, 3 × 10^4^ PFU	Acceleration of colitis development, but no difference in histology	[96]
Allergic airway disease	C57BL/6 mice inoculated intra tracheally	TCV, 10^6^ PFU	Exposed to ovalbumin, CMV-exacerbated disease	[97]

^1^ Salivary gland-derived virus. ^2^ Tissue culture-derived virus. ^3^ Plaque-forming units.

**Table 4:** Mouse models of human congenital cytomegalovirus (cCMV) disease (chronological order).

Inoculation Details	Mouse	SGV ^1^/TCV ^2^ PFU ^3^	Effect	Year	Reference
Inoculation of pregnant mice (approx.) Day 8 Inoculation of fetal and neonatal mice	Outbred Harvard	1000 fifty-percent tissue culture doses (stock was stored as SGV)	Placental infection, but no evidence of fetal infection after maternal infection. Fetal infection (often lethal) shows that fetal mice are susceptible.	1969	[122]
Pregnant mice inoculated i.p. or intramuscularly on E_8_	TO Swiss outbred and BALB/c	SGV 1.1 × 10^7^ (Infective Dose_50_) at various concentrations	i.p. administration produced more fetal wastage and smaller litter sizes. Placental weights not changed. Maternal illness pronounced.	1978	[123]
MCMV injected into endometrial lumina on day of implantation	CF1 (Albino Swiss)	TCV, 100–200 PFU	Reduced litter sizes, malformed fetuses (neural tube defects).	1987	[124]
Maternal i.p. inoculation on Day 8 of pregnancy	BALB/c, BALB/K, CBA	SGV, 3.57 × 10^3^ PFU	Dose dependent, effect related to MHC. No fetal infection. Some placental infection.	1991	[125]
In utero inoculation of fetuses, Day 8	BALB/c and CBA	SGV, 11 PFU	High resorption rate in BALB/c.	1991	[125]
Microinjection of fertilized ova, cultured to blastocyst and implanted	F1 SJL × C57BL/6J	2 pL MCMV DNA (2.5 µg/mL)	Maldeveloped fetuses, increased resorptions.	1993	[126,127]
Inoculation of conceptus D8.5	ICR mice	TCV, 10^4^ PFU	Microphthalmia and cerebral atrophy, potentially model of subclinical cCMV.	1995	[126,127]
Intraplacental inoculation of 10 ng TNF-alpha (D12.5), i.v. inoculation of mother 2 h later	ICR mice	TCV, 10^5^ PFU	Some growth retardation and microcephaly (25%).	2000	[128]
Maternal inoculation of SCID mice i.p.	SCID mice	SGV, 10^3^ PFU (varied)	Maximal placental transmission with maternal infection at E4 (i.p.).	2007	[129]
MCMV inoculated i.p. into newborn pups	BALB/c	TCV, 500 PFU	1. Infection in brain. 2.P3–P5 prednisolone cochlear inflammation.	2008 2019	[130], reviewed in [131] [132]
i.p. inoculation of neonatal mice plus intracerebral inoculation of *E. coli*-derived lipopolysaccharide	BALB/c mice	TCV, 10^5^	Labyrinthitis, significant increase in mean sound pressure level responses due to disruption of the blood labyrinth barrier.	2008	[133]
i.p. RM427+ (recombinant virus expressing LacZ from ie2) mated 5–12 days later, evaluated on D17–18 of pregnancy	C57BL/6	TCV, 10^6^	Increased vasodilation in pregnancy implicating CMV in hypertensive disorders.	2009	[134]
Intracerebral inoculation of neonatal mice	BALB/c	TCV, 17–1700 PFU	Sensorineural hearing loss with dose response.	2015	[135]
Acute group inoculated i.p. MCMV at E7.5 pre-pregnancy, inoculated i.p. mated one month later, PBS E12.5	BALB/c mice	SGV, 5 × 10^3^ PFU	Acute toll-like receptor 2 and 4 upregulation in acute group. Increased placental size in pre-pregnancy group.	2018	[136]

^1^ Salivary gland derived virus, ^2^ Tissue Culture derived virus ^3^ Plaque forming Units.

## Abbreviations

MCMV	Murine cytomegalovirus
HCMV	Human cytomegalovirus
SGV	Salivary gland virus
TCV	Tissue culture virus
PFU	Plaque forming unit
i.p.	Intraperitoneal
i.v.	Intravenous
TNF	Tumor necrosis factor
MCP-1	Monocyte Chemoattractant Protein-1
ICAM-1	Intercellular Adhesion Molecule 1
TGF-β	Transforming Growth Factor-beta
CTL	Cytotoxic lymphocytes
PDGF	Platelet-derived growth factor
E0.5	Day of fertilization
